# Cost‐effectiveness analysis of the artificial intelligence diagnosis support system for early gastric cancers

**DOI:** 10.1002/deo2.289

**Published:** 2023-08-28

**Authors:** Shion Yonazu, Tsuyoshi Ozawa, Tamiji Nakanishi, Kentaro Ochiai, Junichi Shibata, Hiroyuki Osawa, Toshiaki Hirasawa, Yusuke Kato, Hisao Tajiri, Tomohiro Tada

**Affiliations:** ^1^ Faculty of Medicine The University of Tokyo Tokyo Japan; ^2^ AI Medical Service Inc. Tokyo Japan; ^3^ Tada Tomohiro Institute of Gastroenterology and Proctology Saitama Japan; ^4^ Department of Surgical Oncology, Graduate School of Medicine The University of Tokyo Tokyo Japan; ^5^ Departments of Medicine and Gastroenterology Division of Gastroenterology, Jichi Medical University Tochigi Japan; ^6^ Department of Gastroenterology Cancer Institute Hospital of the Japanese Foundation for Cancer Research Tokyo Japan; ^7^ Department of Innovative Interventional Endoscopy Research The Jikei University School of Medicine Tokyo Japan

**Keywords:** artificial intelligence, computer‐aided diagnosis, cost‐effectiveness, early gastric cancer, medical cost

## Abstract

**Objectives:**

The introduction of artificial intelligence into the medical field has improved the diagnostic capabilities of physicians. However, few studies have analyzed the economic impact of employing artificial intelligence technologies in the clinical environment. This study evaluated the cost‐effectiveness of a computer‐assisted diagnosis (CADx) system designed to support clinicians in differentiating early gastric cancers from non‐cancerous lesions in Japan, where the universal health insurance system was introduced.

**Methods:**

The target population to be used for the CADx was estimated as those with moderate to severe gastritis caused by *Helicobacter pylori* infection. Decision trees with Markov models were built to analyze the cumulative cost‐effectiveness of using CADx relative to the pre‐artificial intelligence status quo, a condition reconstructed from data in published reports. After conducting a base‐case analysis, we performed sensitivity analyses by modifying several parameters. The primary outcome was the incremental cost‐effectiveness ratio.

**Results:**

Compared with the status quo as represented in the base‐case analysis, the incremental cost‐effectiveness ratio of CADx in the Japanese market was forecasted to be 11,093 USD per quality‐adjusted life year. The sensitivity analyses demonstrated that the expected incremental cost‐effectiveness ratios were within the willingness‐to‐pay threshold of 50,000 USD per quality‐adjusted life year when the cost of the CAD was less than 104 USD.

**Conclusions:**

Using CADx for EGCs may decrease their misdiagnosis, contributing to improved cost‐effectiveness in Japan.

## INTRODUCTION

Gastric cancer is a significant cause of cancer‐related morbidity and mortality worldwide. In 2020, gastric cancer was the fifth most common cancer and the third most common cause of cancer‐related deaths.^1^ Unlike for late‐stage diagnosis, the prognosis of gastric cancer is relatively good if detected at an early stage. The Japanese gastric cancer screening guidelines recommend endoscopic screening to reduce gastric cancer‐related mortality.^2^ The medical and economic value of this effort has been demonstrated in various global reports.^3^, ^4^

Despite the successes of endoscopic gastric cancer screening, several challenges remain. For one, even skilled endoscopists face difficulty detecting and differentiating cancerous gastric lesions. Most early‐stage gastric cancers (EGCs) occur in sites of atrophic gastritis caused by *Helicobacter pylori* infection. Thus, these neoplastic lesions are challenging to detect because the gastric mucosa reveals only minute changes that can be mistaken as inflammatory changes. Furthermore, wide disparities remain in the diagnostic abilities of endoscopists, and not all patients have access to medical facilities in which gastric cancer screening is conducted.

A computer‐assisted diagnosis support system (CADx) can potentially resolve these issues.^5^ Recently, Ishioka et al. developed a novel artificial intelligence (AI)‐based CADx for EGCs, named Tango.^6^, ^7^ Tango analyzes images acquired during endoscopic procedures to differentiate suspected epithelial neoplastic lesions (e.g., adenoma and adenocarcinoma). According to this previous study, the performance of the AI‐based diagnosis support system was equivalent or superior to that of endoscopic specialists in differentiating EGCs. Based on the result of this study, Tango may soon be introduced in clinical practices in Japan.

However, both the effectiveness and cost of a screening method should be considered. To the best of our knowledge, no study has analyzed the cost‐effectiveness of AI‐based diagnosis support tools for gastric cancer.

Therefore, we analyzed the cost‐effectiveness of the CADx for EGCs based on the results of the previous studies.^8^

## METHODS

### Target population

We targeted cases that were likely to undergo upper gastrointestinal (endoscopy aided by Tango during a given year. These were cases in which the endoscopists identified a possible gastric neoplasm but were unable to independently determine whether the lesion was benign or not, based on the endoscopic findings (i.e., cases with potential EGCs). It was assumed that the upper gastrointestinal endoscopies were conducted with the appropriate health insurance coverage. No published findings regarding the frequency and distribution of such cases are currently available. Therefore, we estimated the target population based on the fact that most gastric cancers are associated with *H. pylori* infection.^9^, ^10^ We also considered the results of a previous study, which demonstrated that the risk of gastric cancer could be stratified by the extent of atrophic gastritis, which can be endoscopically estimated among a population with *H. pylori* infection. The risk is relatively high in patients with moderate to severe atrophic gastritis (from C3 to O3 in the Kimura‐Takemoto classification), with a gastric cancer incidence rate of >2.57%. However, the incidence rate is relatively low (0.11%) in those with mild atrophic gastritis (C1–C2).^11^ Considering these studies, we defined the potential target population of this study, who were likely to undergo a Tango diagnosis during upper endoscopy, as those who had moderate to severe atrophic gastritis caused by *H. Pylori* infection (Figure S1).

### Model

Patients who underwent endoscopy were categorized into the following four groups based on the relationship between the ‘true’ diagnosis, which was based on the pathological findings, and the diagnosis assessed by the endoscopist during the procedure: 1) non‐neoplastic case endoscopically diagnosed to be non‐neoplastic (TN: true negative), 2) non‐neoplastic cases endoscopically diagnosed to be neoplastic (FP: false positive), 3) neoplastic case endoscopically diagnosed to be neoplastic (TP: true positive), and 4) neoplastic case endoscopically diagnosed to be non‐neoplastic (FN: false negative).

**FIGURE 1:**
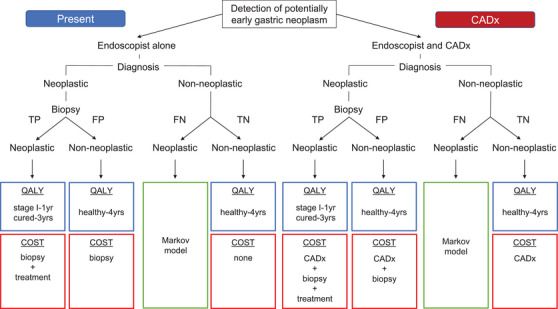
The quality‐adjusted life years (QALYs) and medical costs for the present and the computer‐assisted diagnosis (CADx)‐use cases. For the false negative cases, the Markov model was applied to simulate the QALY and medical costs.

In FP and TP endoscopic examinations, biopsies were conducted to confirm or deny the endoscopist's suspicion of a neoplasm. The FN group received no further diagnostic or treatment at baseline and was included in the Markov model as shown in Figure 1. Any gastric cancer misdiagnosed at the baseline endoscopic examination was considered a stage I lesion because advanced cancers are rarely misdiagnosed. In patients with misdiagnosed gastric cancers, three distinct possibilities were considered one year after the baseline examination: no cancer progression, cancer progression to the later stages, or death.

Estimates of the status quo regarding gastric cancer screening were made based on the National Database (NDB) of Japan (https://www.mhlw.go.jp/stf/seisakunitsuite/bunya/0000177221_00010.html.) and other published reports, which reflected the Japanese data.^12^ This pre‐AI scenario was compared to the hypothetical scenario in which Tango was widely introduced into the clinical environment.

To establish an endpoint for the study, we assumed that the second endoscopic examination would be performed 3 years after the first examination for the total population, and we set the analysis period as 3 years.^13^

### Assumptions

We assumed that all patients in the FP and TP groups underwent biopsy, and the accuracy of biopsies was hypothetically set as 100%. In the FN group, it was assumed that all neoplasms were diagnosed at the second endoscopic examination 3 years later. Furthermore, we assumed that all neoplasms misdiagnosed at the initial endoscopic examination remained undiagnosed before the aforementioned second endoscopy.

Irrespective of initial categorization, it was assumed that all patients received appropriate and necessary treatments when their gastric cancers were diagnosed.

### Parameters

According to the NDB data, 8,585,843 endoscopies were performed under the Japanese public insurance system from April 2019 to March 2020.

As described above, the target population for Tango could be approximated to those with moderate to severe atrophy caused by *H. pylori* infection. Based on a previous study, we assumed that *H. pylori* infection was present in about 40% of patients undergoing endoscopy through the public insurance system.^14^ We subsequently assumed that the moderate to severe atrophy rate was 65.4% in this population.^11^ Therefore, it was estimated that Tango might be applied to approximately 2.2 million endoscopic cases annually (Figure S1).

The number of gastric cancers detected in Japan in 2019 was 124,319 based on the Japanese cancer registry (https://www.mhlw.go.jp/content/10900000/000942181.pdf.) and the ratio of EGCs (stage I) detected in 2020 was 59.6% based on Japanese cancer registry.^14^, ^15^ Therefore, the number of EGCs diagnosed in a year was estimated to be 74,094. Previous studies demonstrated that EGCs are missed or misdiagnosed with an FN rate of 25.8%; consequently, it could be assumed that 25,763 EGCs were undiagnosed annually.^13^

The average biopsy rate during the endoscopy was set as 20% ​according to a private survey of Japanese endoscopists and Japanese endoscopic screening manuals for gastric cancer.^16^

Because most biopsies for histologically proven benign lesions (FP cases) would be endoscopically diagnosed as potential EGCs and not as advanced cancers, the annual number of biopsies for the identification of EGCs was calculated as [(total number of biopsies taken) – (annual number of advanced gastric cancers) – (annual number of esophageal cancers)] (Figure S2). The annual number of esophageal cancers was estimated as 25,000 cases according to the Japanese cancer registry (https://jhcr‐cs.ganjoho.jp/hbcrtables/wpCancerSearch.aspx?UnitType=2.)^15^

The diagnostic performances of Tango for the target population were assumed to be the same as that in the previous study (sensitivity of 84.7% and specificity of 58.2%; 8).

When calculating health outcomes, the probability of death within 1 year was calculated based on the 5‐year survival rates of Cancer Survival in Hospital‐based Cancer Registries (https://www.mhlw.go.jp/content/10900000/000942181.pdf.).^17^ The probabilities of progression from Stage I to II, Stage II to III, and Stage III to IV were set as 0.035 (0.002–0.068), 0.192 (0.123–0.261), and 0.393 (0.393), respectively, based on the data used in a Japanese microsimulation.^18^ Note that the transition probabilities for Stages I to II were decided based on the clinical probabilities of progression from dysplasia to local preclinical cancer. In addition, the base case was set to the median of the parameter range of probabilities obtained from previous studies.^18^

**FIGURE 2:**
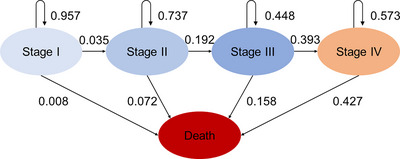
The probabilities of progression and morbidity of gastric cancer in each stage.

The probability of non‐progression (i.e., remaining in the current stage) was calculated as [1 − (probability of progressing to the later stage) – (probability of death)]. Note that Stage IV was the final stage, and no further progression existed (Figure 2).

The quality‐adjusted life years (QALYs), calculated as one year of life multiplied by a utility factor ranging from 0 (death) to 1 (healthy), was set for each stage of gastric cancer as 0.90 (stage I), 0.81 (stage II), 0.69 (stage III), and 0.47 (stage IV), respectively, according to the previous studies.^19^, ^20^ QALYs after full recovery with appropriate interventions (defined as ‘cured’) were set as 0.95.^19^, ^20^

### Costs

Medical costs were examined only from the perspective of the medical payer.

Based on a previous study, treatment costs in Japan for Stages I, II, III, and IV were fixed at 7464, 14,402, 22,736, and 195,742 USD, respectively.^20^ The cost of the biopsy and pathological analysis was set at 80 USD in Japan.

We estimated that the installation and annual contract costs of Tango would be 6000 and 10,000 USD, respectively. Assuming that Tango would be used for 250 cases annually (if 1000 upper endoscopies are performed annually, and Tango is utilized in 25% of them) over five consecutive years, the per‐endoscopy cost of Tango would be 44.8 USD. Therefore, we estimated the marginal cost of using Tango was 45 USD. In a case where a biopsy is performed after an endoscopy, the use of Tango is not billed to insurance.

All costs were converted to USD using the exchange rate on July 1, 2023 (1 USD = 140 JPY) as reported by the Organization for Economic Cooperation and Development.

### Outcome measures

The primary outcome of this study was the incremental cost‐effectiveness ratio (ICER). ICER represents the marginal endoscopy‐related costs required to achieve a marginal increase of 1 QALY. The willingness‐to‐pay threshold (WTP) was set at 50,000 USD, and ICER ≤ 50,000 USD/QALY was considered cost‐effective.^21^

First, we conducted a base case analysis that incorporated the baseline variables shown in Table 1. Thereafter, we evaluated outcome variability and conducted sensitivity analyses for the probability of death, disease progression, QALYs, and the costs of treatment for each cancer stage. We also determined the probability of biopsy and undiagnosed cases of EGC, as well as the sensitivity and specificity of Tango itself (Table 1). A second probabilistic sensitivity analysis was conducted with a Monte Carlo simulation to investigate the effect of parameter uncertainty on the cost‐effectiveness results. The model was run 500 times, each taking random draws from all inputs with the prespecified uncertainty distributions listed in Table 1. In accordance with the Japanese Guidelines for the Economic Evaluation of Health Insurance Technologies, costs, and QALYs were discounted at an annual rate of 2%.

**TABLE 1:** Details of the parameters used in the base‐case and sensitivity analyses.

Parameters	Base	Range
Endoscopy	8,585,843	‐
*H. Pylori* infection	0.400	‐
CADx use (rate)	0.262	0.208–0.323
Biopsy rate	0.200	0.139–0.220
Gastric cancer		
Total	124,319	‐
Early	74,094	‐
Miss‐diagnosis rate of EGC	0.258	0.227–0.291
Stage I		
No progression	0.957	0.925–0.989
Progression (I–II)	0.035	0.002–0.068
Mortality	0.008	0.008–0.009
Stage II		
No progression	0.737	0.672–0.804
Progression (II–III)	0.192	0.123–0.261
Mortality	0.072	0.067–0.073
Stage III		
No progression	0.448	0.444‐0.452
Progression (III–IV)	0.393	0.300‐0.393
Mortality	0.158	0.155–0.162
Stage IV		
No progression	0.573	0.568–0.581
Progression	0	‐
Mortality	0.427	0.432–0.419
QALY		
Healthy	1.00	‐
Cured	0.95	0.90–1.00
Stage I	0.90	0.85–0.95
Stage II	0.81	0.76–0.86
Stage III	0.69	0.64–0.74
Stage IV	0.47	0.42–0.52
Death	0	‐
Cost		
Biopsy	80	‐
CADx	45	15–110
Treatment		
Stage I	7464	3732–14,928
Stage II	14,402	7201–28,804
Stage III	22,736	11,368–45,472
Stage IV	195,742	97,871–391,484
Performances of CADx		
Sensitivity	0.847	0.787–0.900
Specificity	0.582	0.509–0.655
Discount rate	0.020	‐

Abbreviations: CADx, computer‐assisted diagnosis; EGC, early gastric cancer; QALY, quality‐adjusted life year.

All analyses were performed using TreeAge Pro 2022 software.

## RESULTS

### Incremental cost‐effectiveness ratio of the base case

In the present situation, the estimated number of patients of TP, FP, FN, and TN cases were 74,094 (3.3%), 1567,849 (69.8%), 25,763 (1.1%), and 578,350 (25.7%), respectively. While, in the CAX use situation, the estimated number of patients of TP, FP, FN, and TN cases were 84,579 (3.8%), 897,111 (39.9%), 15,278 (0.7%), and 1249,088 (55.6%), respectively.

The estimated number of patients with GC of each stage at the second endoscopy and the number of patients deceased during the 3 years were 22,580 (stage I), 1,951 (stage II), 370 (stage III), 68 (stage IV), and 794 (death) in the present situation. While, those numbers were 13,390 (stage I), 1157 (stage II), 220 (stage III), 40 (stage IV), and 471 (death) in the CADx use situation.

The estimated total medical cost of the current practice was 902,634,164 USD, which increased to 940,306,450 USD if the CADx was utilized. Thus, the incremental cost was calculated as –37,672,286 USD.

Compared to the present situation, the total incremental effectiveness was calculated as 3396 QALY, and the ICER was estimated as 11,093 USD/QALY.

### Sensitivity analyses

**FIGURE 3:**
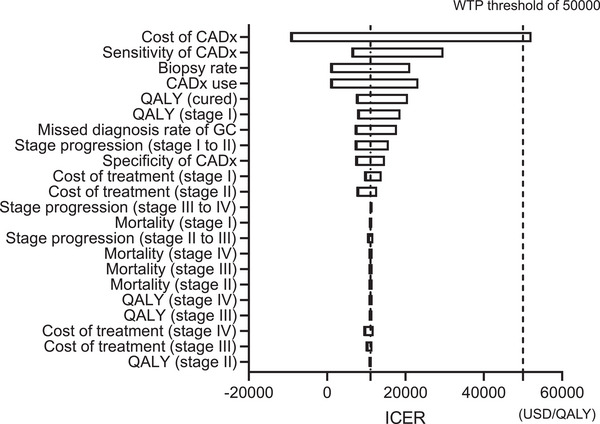
A tornado diagram representing the results of the one‐way sensitivity analysis.

A Tornado diagram representing the results of the one‐way sensitivity analysis is shown in Figure 3. The results of the sensitivity analysis showed that the ICERs associated with Tango adoption and use did not exceed the WTP threshold of 50,000 USD in all the expected cases when the cost of CADx was less than 104 USD.

In addition, probabilistic sensitivity analysis was performed using Monte Carlo simulations, and the expected ICERs in all cases were within the WTP threshold of 50,000 USD.

## DISCUSSIONS

A previous study showed that the sensitivity of the endoscopic CADx system to diagnose EGCs was superior to that of endoscopic specialists and that it would reduce EGC misdiagnosis in clinical settings. Detecting gastric cancers early may reduce medical costs, including hospital admission, and surgical and chemotherapy fees.

However, the specificity of the CADx system was lower than that of endoscopists, which could lead to an increased number of biopsies taken under the CADx system, resulting in additional medical costs. Considering these issues, we, for the first time, evaluated the cost‐effectiveness of the CADx system for EGCs.

Our results indicated that the adoption and use of the AI‐based endoscopic CADx could result in an ICER value of 11,093 USD/QALY. Assuming that Tango is implemented in all institutions and is utilized in cases with moderate to severe atrophic gastritis caused by *H. pylori* in Japan. Additionally, the sensitivity analyses conducted by modifying several parameters showed that the expected results were predicted to be below the WTP threshold of 50,000 USD/QALY in any case, when the cost of the CADx was less than 104 USD.

Thus, the introduction of the AI‐based endoscopic CADx for EGC is expected to be clinically valuable for supporting endoscopists in EGC diagnosis and for saving costs in Japan.

Regarding endoscopic CAD systems, several AI‐based CAD systems for colonoscopy have been developed. Most are CAD systems for detecting colorectal polyps or diagnosing colorectal neoplasms. These systems increased the adenoma detection rate in several randomized studies and are already used in actual clinical settings. In addition, Mori et al. demonstrated that using a computer‐assisted detection system for colorectal polyps may reduce medical costs by decreasing the incidence of colorectal cancers, although it may increase the incidence of polypectomy.^22^ Sekiguchi et al. evaluated the cost‐effectiveness of utilizing a CAD system for detecting colorectal polyps in the context of the Japanese colorectal cancer screening system and concluded that the CAD had the potential to be cost‐effective.^23^

Regarding cost‐effectiveness in gastric cancer, Huang et al. analyzed the cost‐effectiveness of endoscopic gastric cancer screening. They developed a microsimulation model representing the Japanese population and evaluated 15 endoscopic screening scenarios. Using the same thresholds as in the current study, they found that a triennial screening program for individuals aged 50 to 75 years was cost‐effective.^18^

The cost‐effectiveness of this type of AI‐based diagnosis support tool may be the same in many other countries, but further studies are needed because epidemiology, medical costs, and other factors differ from country to country, especially those associated with gastric cancer.

There are several limitations to this study. First, the study was based on a previous study that used still endoscopic images selected for the performance comparison. The sensitivity and specificity were evaluated with the image‐based analysis and not with the patient‐based analysis. As described in the limitations of the previous study our research was based on, the distribution of the included cases was different from actual clinical settings, and the performances of the CADx system may also be different when applied in actual clinical practices.^8^ Therefore, this economic analysis can be refined in the future. Second, this simulation was based on data from previous studies, and the results of this study may vary according to the utilized data. Furthermore, direct data on several variables were unavailable, and we extrapolated and estimated the value of these variables to the maximum extent possible given the available findings.

To minimize the effects of these limitations, we conducted several sensitivity analyses that utilized modifications of several variables, and the results still demonstrated the cost‐effectiveness of the CADx system. However, we emphasize that this study is a simulation model under an ideal setting, and we expect that the cost‐effectiveness of the CADx system will be revealed in real clinical settings in the near future.

In conclusion, the examined AI‐based diagnosis support tool for EGCs demonstrated fundamental cost‐effectiveness in Japan.

## CONFLICT OF INTEREST STATEMENT

Tomohiro Tada and Yusuke Kato are the CEO and the CTO of AI Medical Service Inc., respectively. Tsuyoshi Ozawa, Junichi Shibata, and Kentaro Ochiai are consulting members of AI Medical Service Inc. Hisao Tajiri received an advisory fee from AI Medical Service Inc., and lecture fees from Olympus Medical Co. and Fujifilm Medical Co. Hiroyuki Osawa and Hisao Tajiri received an advisory fee from AI Medical Service Inc. Shion Yonazu and Toshiaki Hirasawa have no conflict of interest.

## Supporting information


**Figure S1**: We defined the target population who may be utilized the CADx as those who have moderate to severe atrophic gastritis caused by *H. Pylori* infection.
**Figure S2**: Biopsies that were performed for the identification of early‐stage gastric cancers were calculated as a sum of the true positive and false positive cases.
