# Supplementary material for: Cost‐effectiveness analysis of the artificial intelligence diagnosis support system for early gastric cancers
Source: DEN Open. 2023 Aug 28;4(1):e289. doi: 10.1002/deo2.289 (PMC10461711; doi:10.1002/deo2.289)
Supplement: Supplementary file 1 — Figure S1: We defined the target population who may be utilized the CADx as those who have moderate to severe atrophic gastritis caused by H. Pylori infection. Figure S2: Biopsies that were performed for the identification of early‐stage gastric cancers were calculated as a sum of the true positive and false positive cases. [file DEO2-4-e289-s001.docx]

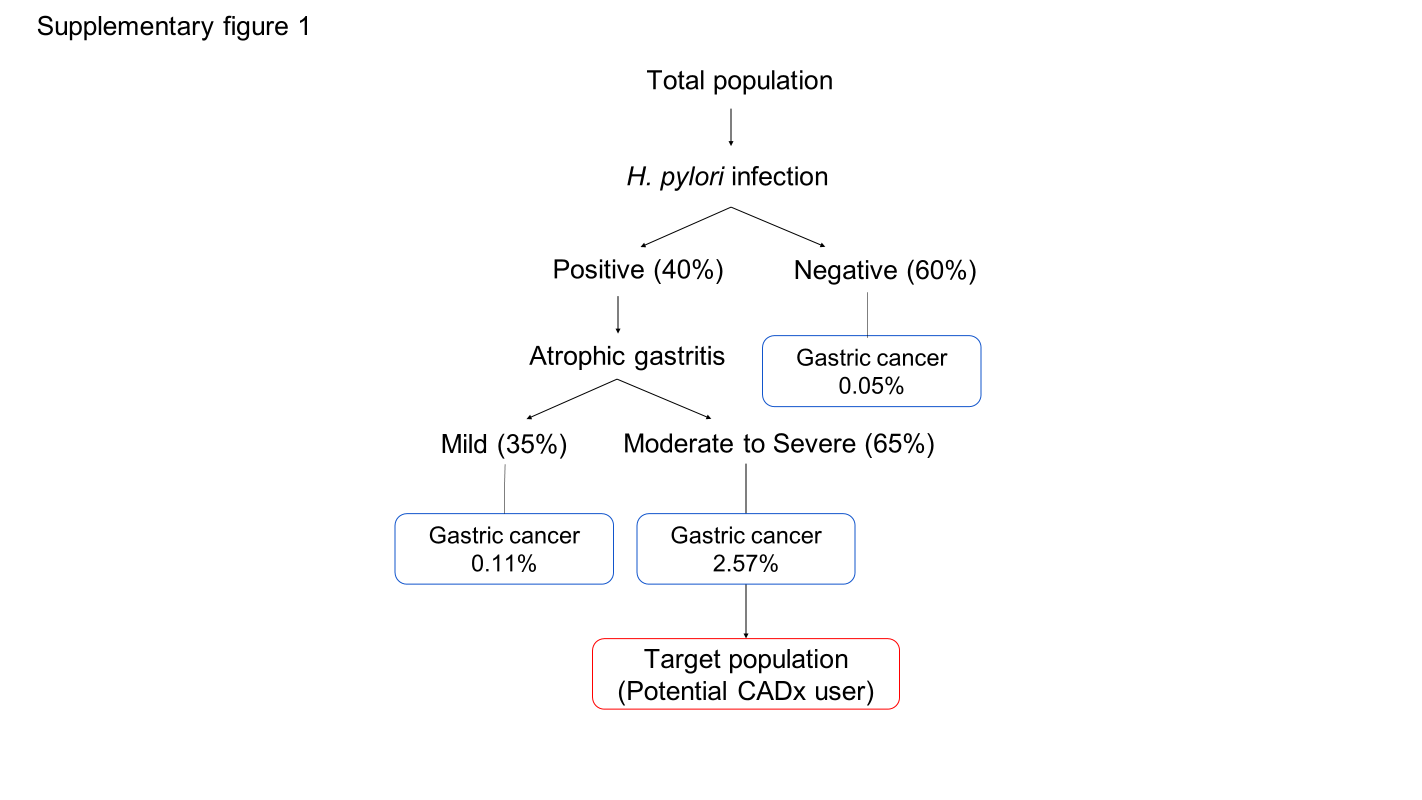


We defined the target population who may be utilized the CADx as those who have moderate to severe atrophic gastritis caused by *H. Pylori* infection.


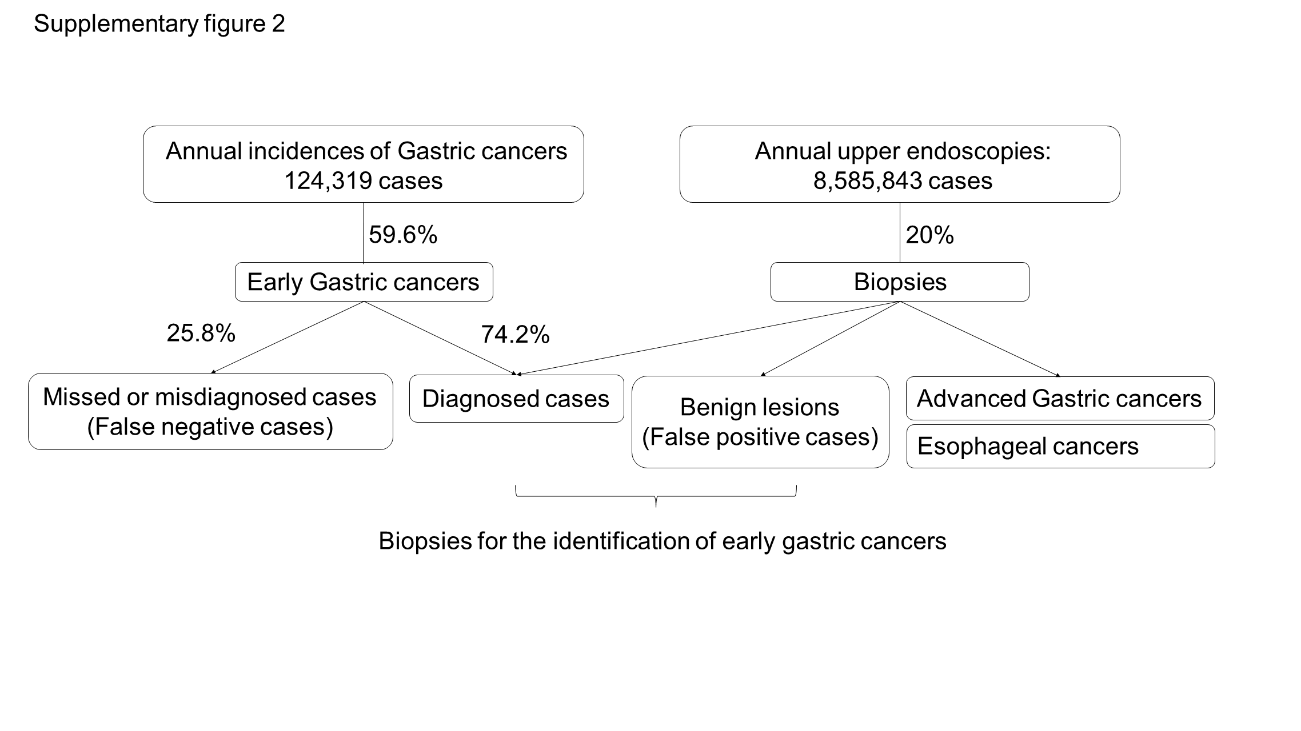


Biopsies which were performed for the identification of early-stage gastric cancers were calculated as a sum of the true positive and false positive cases.
